# Progesterone effects on vaginal cytokines in women with a history of preterm birth

**DOI:** 10.1371/journal.pone.0209346

**Published:** 2018-12-31

**Authors:** David J. Garry, David A. Baker, Malini D. Persad, Tatyana Peresleni, Christina Kocis, Michael Demishev

**Affiliations:** Department of Obstetrics & Gynecology and Reproductive Medicine, Stony Brook Medicine, Stony Brook, NY, United States of America; Case Western Reserve University, UNITED STATES

## Abstract

**Objective:**

To determine the effect of intramuscular progesterone on the vaginal immune response of pregnant women with a history of prior preterm birth.

**Methods:**

A prospective, cohort study of women at 11–16 weeks gestation, ≥18 years of age, and carrying a singleton pregnancy was conducted from June 2016 to August 2017 after IRB approval. Women in the progesterone arm had a history of preterm birth and received weekly intramuscular 17-hydroxyprogesterone caproate. Controls comprised of women with healthy, uncomplicated pregnancies. Excluded were women with vaginitis, diabetes mellitus, hypertension, or other chronic diseases affecting the immune response. A vaginal wash was performed at enrollment, at 26–28 weeks, and at 35–36 weeks gestation. Samples underwent semi-quantitative detection of human inflammatory markers. Immunofluorescence pixel density data was analyzed and a P value <0.05 was considered significant.

**Results:**

There were 39 women included, 10 with a prior preterm birth and 29 controls. The baseline demographics and pregnancy outcomes for both groups were similar in age, parity, race, BMI, gestational age at delivery, mode of delivery, and birth weight. Enrollment cytokines in women with a prior preterm birth, including IL-1 alpha (39.2±25.1% versus 26.1±13.2%; P = 0.04), IL-1 beta (47.9±26.4% versus 24.9±17%; P<0.01), IL-2 (16.7±9.3% versus 11.3±6.3%; P = 0.03), and IL-13 (16.9±12.4% versus 8.2±7.4%; P = 0.01) were significantly elevated compared to controls. In the third trimester the cytokine densities for IL-1 alpha (26.0±18.2% versus 22.3±12.0%; P = 0.49), IL-1 beta (31.8±15.9% versus 33.1±16.8%; P = 0.84), IL-2 (10.0±8.4% versus 10.9±5.9%; P = 0.71), and IL-13 (9.1±5.9% versus 10.0±6.5%; P = 0.71) were all statistically similar between the progesterone arm and controls, respectively.

**Conclusion:**

There is an increased cytokine presence in vaginal washings of women at risk for preterm birth which appears to be modified following the administration of 17- hydroxyprogesterone caproate to levels similar to healthy controls.

## Introduction

Intramuscular progesterone, administered to women at risk of preterm birth, reduces the likelihood of a subsequent preterm birth by approximately one-third [1,2]. The exact mechanism of action of progesterone therapy in preventing preterm birth is not well understood [3]. To date, studies have inadequately investigated progesterone’s mechanisms of action in vivo. Despite the lack of understanding, there has been an increasing use of progesterone in pregnancy for prevention of preterm birth. While several theories exist surrounding the mechanisms of action, which include immunomodulation effects, signaling pathway regulation, and progesterone receptors alteration, the protective effect of progesterone may be manifested through cytokine modification [4].

At the onset of labor, the ratio of progesterone receptor-A (PR-A) to progesterone receptor-B (PR-B) increases. Due to an increase in myometrial PR-A, there is a functional withdrawal of progesterone and an increase in sensitivity to contractile stimuli [3]. This effect is also modulated by prostaglandins produced prior to the onset of labor [3]. Progesterone-dependent immunomodulation is another plausible mechanism that enables pregnancy to proceed to term. Immunologic effects of progesterone are mediated by a 34-kDa protein named progesterone-induced blocking factor (PIBF) [4]. Progesterone-induced blocking factor, synthesized by lymphocytes of healthy pregnant women in the presence of progesterone, inhibits natural killer cell (NK) activity and modifies the cytokine balance [5]. Rodent fetoplacental human artery explant models and experiments with human lymphocytes have shown progesterone to downregulate the immune response [6,7]. Additionally pretreatment with progesterone prior to intrauterine infection has been associated with a decrease in bacteria-induced upregulation of Toll-like receptors in the cervix and placenta [8].

Recently, Monsanto et al compared women with cervical insufficiency with normal controls and demonstrated elevated levels of proinflammatory cytokines in cervicovaginal fluid, suggesting a dysregulation of the local vaginal immune environment [9]. Placement of a cervical cerclage significantly reduced the proinflammatory cytokines, suggesting that cerclage may prevent preterm birth through reduction of local inflammation in cervical insufficiency.

The objective of this study was to evaluate the vaginal immune response of progesterone supplementation in pregnant women at risk for preterm birth compared to normal healthy pregnant controls.

## Materials and methods

A prospective cohort study was conducted at an academic medical center from June 2016 to August 2017 with Stony Brook University Institutional Review Board (IRB) approval (CORHIS #2016-3441-F). Women were included if they were at a gestational age of 11 to 16 weeks, 18 years of age or older, and carrying a singleton pregnancy without acute or chronic vaginitis, diabetes mellitus, hypertension, and other chronic diseases or therapies that would affect the inflammatory or immune response. Women in the progesterone arm had a history of preterm birth and therefore were candidates for intramuscular 17-hydroxyprogesterone caproate (17-OHP). Controls were comprised of women with healthy uncomplicated pregnancies who did not receive progesterone therapy. All women were recruited prior to initiation of progesterone treatment. Intramuscular progesterone treatment was started at 16–20 weeks gestation in women with a prior preterm birth.

A vaginal rinse was performed at three points in the study: on enrollment into the study at 11 to 16 weeks, at 26 to 28 weeks, and at 35 to 36 weeks gestation. All vaginal rinse samples were collected in subjects while in the supine position with a sterile speculum. A 10 ml syringe filled with 7 ml of sterile water attached to a soft plastic 14-gauge catheter was used to irrigate the vaginal vault. A sterile cotton swab was used to gently rub the mucosal surfaces. The fluid was aspirated back into a sterile 15 ml test tube. The samples were placed on ice and processed within 2 hours [10].

The specimen tube was centrifuged at 3,000 RPM for 10 minutes. Aliquots of the vaginal lavage supernatant were stored at –80 degrees centigrade. The aliquots were later analyzed using Ray-Bio Human Inflammation Antibody Array C3 8-well plates (RayBiotech, Inc., Norcross, GA) according to manufacturer’s instructions. Samples underwent semi-quantitative detection of 40 human inflammatory markers, and all cytokines were evaluated in duplicates. After treatment with biotinylated antibodies, followed by HRP-Streptavidin-labeled antibodies, the chemiluminescence detection of the products was performed using X-ray films. Numerical densitometry data were extracted by NIH software program Image Jv1.51j8 (National Institutes of Health, USA), using a dot array analysis plugin, as described in a previous publication [10]. All images were acquired using the same microscope settings, including filter and exposure parameters, with image acquisition performed from samples processed side by side [11].

Patient demographics including age, parity, ethnicity, pre-pregnancy body mass index (BMI), and tobacco use, were extracted from the medical record. Pregnancy outcomes collected included gestational age at delivery, mode of delivery, birth weight, Apgar scores and Neonatal Intensive Care Unit (NICU) admission.

Categorical variables were analyzed using, Chi square, Fisher’s exact test and continuous, normally distributed variables were analyzed using student’s t test. Cytokine data were normalized by dividing samples’ pixel density by pixel density of a positive control [10,11]. A P value <0.05 was deemed statistically significant. Statistical analysis was performed using SPSS software (IBM SPSS Statistics for Windows, Version 22.0. Armonk, NY).

## Results

Thirty-nine women were included in the evaluation: 10 women with a previous preterm birth utilizing progesterone and 29 healthy control women. The baseline demographics and pregnancy outcomes of both groups were similar in age, parity, race, body mass index (BMI), gestational age at delivery, mode of delivery, and birth weight. (Table 1) Overall, there were three late preterm deliveries, one in the progesterone arm and two in the control arm (10% versus 6.9%; P = 1.0). No women delivered at less than 34 weeks.

**Table 1:** Demographics and pregnancy outcomes.

Demographics	Progesterone (N = 10)	Control (N = 29)	P value
Maternal age (y)	32.3 ± 4.5	29.8 ± 5.1	0.18
Age >35 years old	3 (30.0)	7 (24.1)	0.70
Race/ethnicity			0.20
Caucasian	6 (60.0)	18 (62.0)	
African American	0 (0)	4 (13.8)	
Asian	10 (0)	3 (10.3)	
Hispanic	4 (40.0)	4 (13.8)	
Pre-pregnancy BMI (kg/m^2^)	31.9 ± 7.1	28.2 ± 6.6	0.15
BMI ≥ 30	5 (50.0)	10 (34.5)	0.38
Multiparity	10 (100.0)	20 (69.0)	0.08
Gestational diabetes	2 (20.0)	2 (6.9)	0.27
Smoker	0 (0)	3 (10.3)	0.56
Pregnancy Outcomes			
GA at delivery (wk)	38.8 ± 1.5	39.4 ± 1.4	0.28
Cesarean delivery	3 (30.0)	10 (34.5)	1.00
Delivery < 37 wk	1 (10.0)	2 (6.9)	1.00
Birth weight (g)	3282.6 ± 358.5	3431.5 ± 448.0	0.35
Apgar score at 5 minutes < 7	1 (3.4)	0 (0)	1.00
NICU Admission	2 (20.0)	5 (17.2)	1.00

BMI, body mass index; GA, gestational age; NICU, Neonatal Intensive Care Unit

Data presented as n (%) or mean ± SD

**Fig 1:**
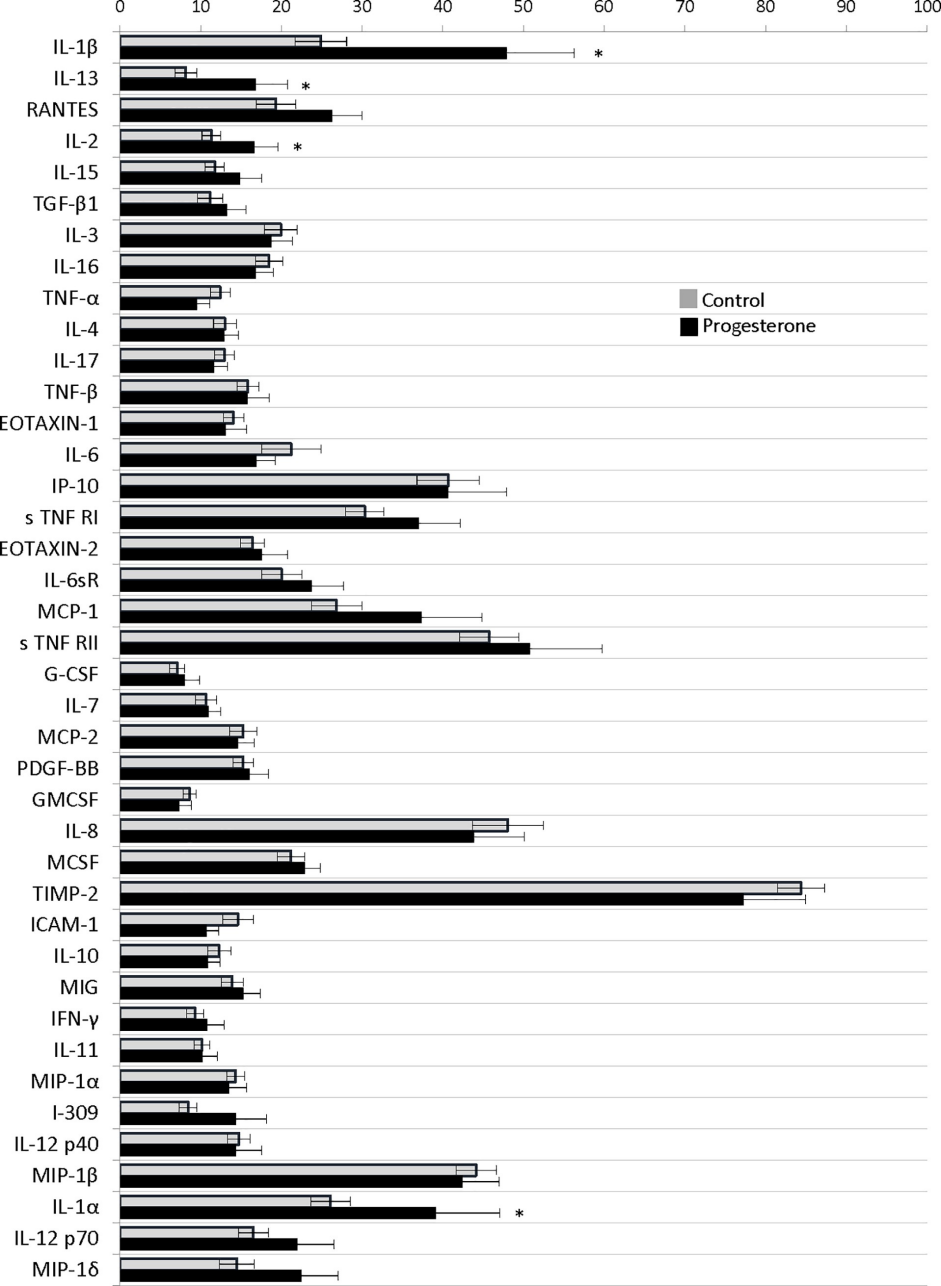
Initial visit cytokines comparison between women with a preterm birth and healthy controls. Complete cytokine profile from initial evaluation at 11 to 16 weeks (visit 1). Data presented as pixel density percentage with * signifying P<0.05.

On initial evaluation at 11 to 16 weeks (visit 1), there were significant differences between inflammatory markers pixel densities in women with a history of preterm birth (progesterone arm) compared with healthy controls. Cytokines from women with a preterm birth including interleukin-1 alpha (39.2±25.1% versus 26.1±13.2%; P = 0.04), interleukin-1 beta (47.9±26.4% versus 24.9±17%; P<0.01), interleukin-2 (16.7±9.3% versus 11.3±6.3%; P = 0.03), and interleukin-13 (16.9±12.4% versus 8.2±7.4%; P = 0.01) were significantly elevated compared to controls (data as preterm birth versus healthy control pixel density percentage, respectively). (Fig 1)

**Fig 2:**
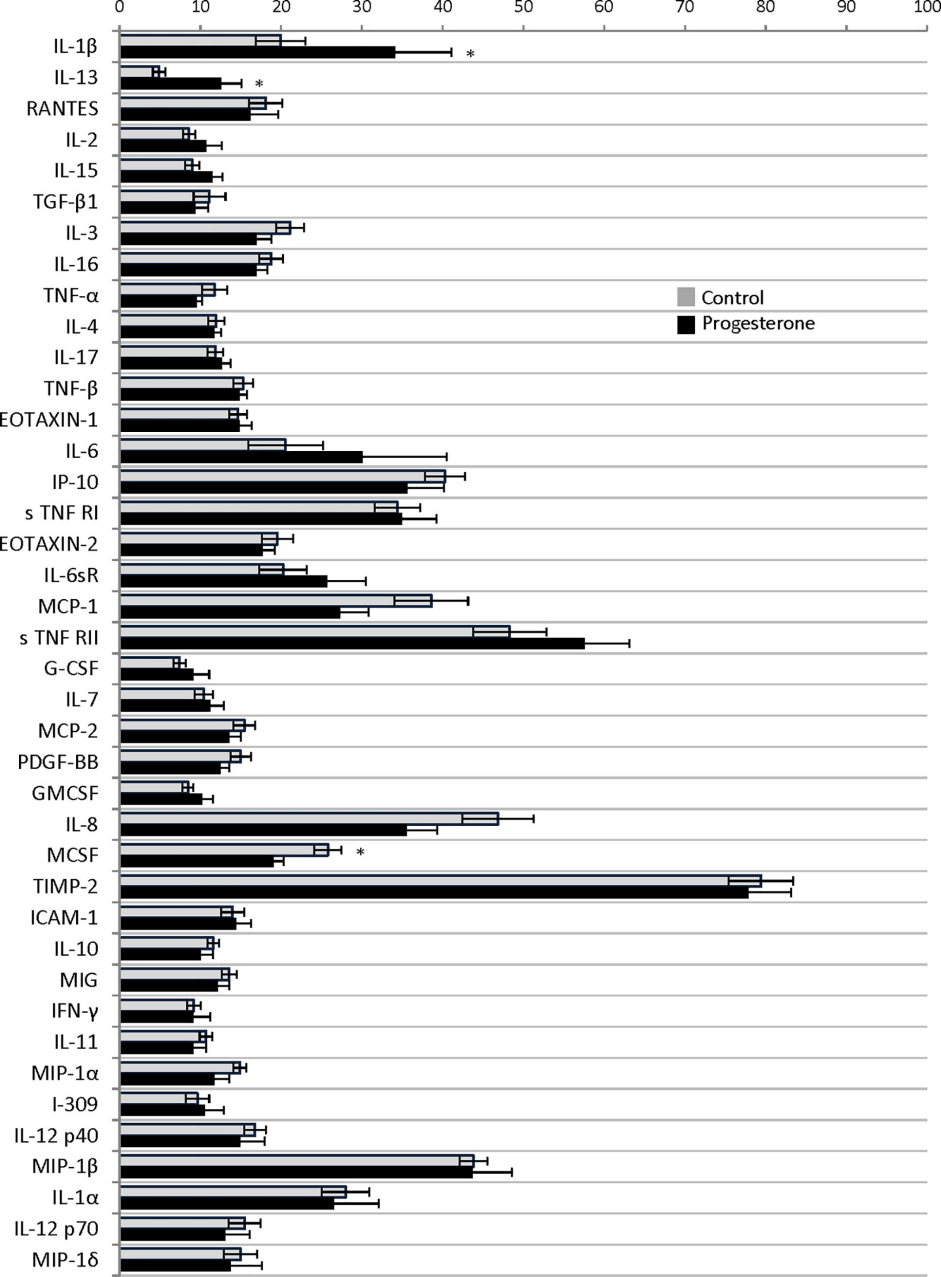
Cytokines comparison between women with a preterm birth including and healthy controls at 26 to 28 weeks. Complete cytokine profile from at 26 to 28 weeks (visit 2). Data presented as density percentage with * signifying P<0.05.

**Fig 3:**
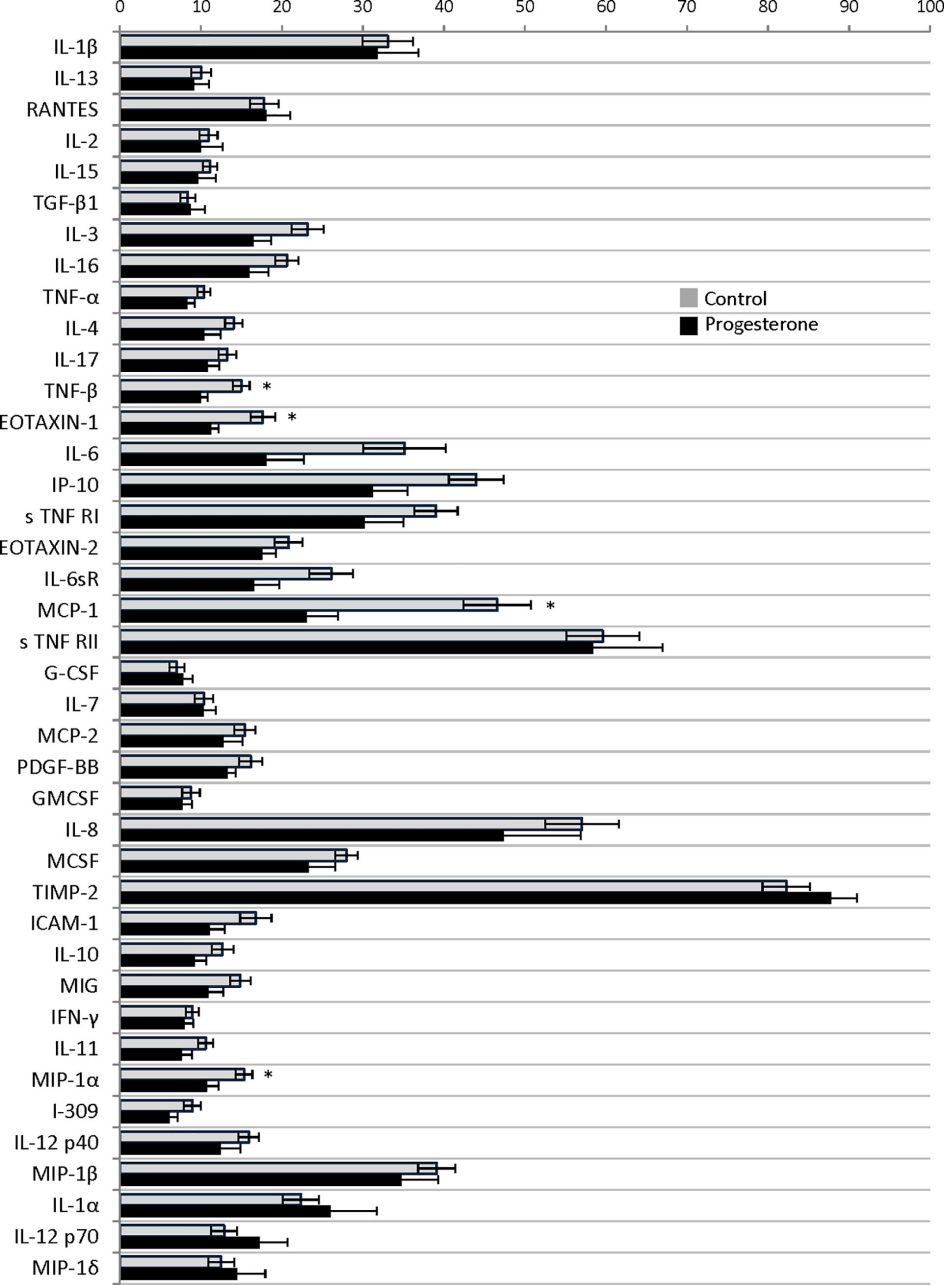
Cytokines comparison between women with a preterm birth including and healthy controls 35 to 36 weeks. Complete cytokine profile from at 35 to 36 weeks (visit 3). Data presented as density percentage with * signifying P<0.05.

Evaluation of women in the progesterone arm in the early third trimester, 26 to 28 weeks (visit 2), demonstrated persistence of significantly elevated interleukin-1 beta (34.2±21.9% versus 19.9±16.5%; P = 0.04) and interleukin-13 (12.6±8.1% versus 4.9±4.3%; P<0.01) when compared with healthy women. Interestingly, interleukin-1 alpha (26.6±17.5% versus 28.0+15.7%; P = 0.81), and interleukin-2 (10.7±6.1% versus 8.6±3.9%; P = 0.22) were statistically similar in both groups. (data as preterm birth versus healthy control pixel density percentage; respectively). In the later third trimester, 35 to 36 weeks (visit 3), the cytokine densities for interleukin-1 alpha (26.0±18.2% versus 22.3±12.0%; P = 0.49), interleukin-1 beta (31.8±15.9% versus 33.1±16.8%; P = 0.84), interleukin-2 (10.0±8.4% versus 10.9±5.9%; P = 0.71), and interleukin-13 (9.1+5.9% versus 10.0+6.5%; P = 0.71) were all statistically similar between the progesterone arm and the healthy controls, respectively. (Figs 2 and 3)

Evaluation of other vaginal cytokines densities in the later third trimester, including tumor necrosis factor beta (TNF-beta) (9.9±2.6% versus 14.9±5.6%; P = 0.01), macrophage inflammatory protein-1 alpha (MIP-1 alpha) (10.8±4.4% versus 15.3±5.6%; P = 0.03), eotaxin-1 (11.3±2.8% versus 17.6±8.3%; P = 0.03), and monocyte chemoattractant protein-1 (MCP-1) (23.1±12.1% versus 46.6±22.4%; P<0.01) showed a significant downregulation when compared to controls. (Table 2) On enrollment and at the 26 to 28 week visit the densities were similar between groups for all four of these cytokines. In women receiving progesterone, TNF-beta, MIP-1 alpha, eotaxin-1 and MCP-1 sequentially decreased at each sampling time; however, this reduction did not reach significance. (Table 2) In the healthy controls, TNF-beta, MIP-1 alpha, and eotaxin-1 densities remained similar throughout the sampling visits. MCP-1 increased in the control group from enrollment (26.9±16.8%) compared with visit 3 at 35 to 36 weeks (46.6±22.4%; P<0.01). (Table 3) All comparisons are made available in Supporting Information. (S1 Table)

**Table 2:** Comparison of cytokines between different visits within progesterone group.

Cytokine	Visit 1 N = 10	Visit 2 N = 10	Visit 3 N = 10
IL-1α	39.2 ±25.1	26.6 ±17.5	26.0 ±18.2
IL-1β	47.9 ±26.4	34.2 ±21.9	31.8 ±15.9
IL-2	16.7 ±9.3	10.7 ±6.1	10.0 ±8.4
IL-6	16.9 ±7.4	30.1 ± 33.0	18.1 ±14.5
IL-13	16.9 ±12.4	12.6 ±8.1	9.1 ±5.9
MCSF	22.9 ±6.2	19.1 ±3.9**	23.3 ±10.4
TNF-α	9.6 ± 5.0	9.5 ±2.2	8.3 ±2.9
TNF-β	15.8 ±8.4	14.9 ±2.8	9.9 ±2.6
MCP-1	37.4 ± 23.5	27.3 ±11.2	23.1 ±12.1
MIP-1α	13.5 ±7.1	11.7 ±6.0	10.8 ±4.4
Eotaxin-1	13.1 ±8.3	14.9 ±4.8	11.3 ±2.8

**P = 0.01 Visit 1 compared to Visit 2

All comparisons are otherwise non-significant (P≥0.05)

IL, interleukin; MCSF, macrophage colony-stimulating factor; TNF, tumor necrosis factor; MCP, monocyte chemoattractant protein; MIP, macrophage inflammatory protein.

Data presented as pixel density percentage ± SD

**Table 3:** Comparison of cytokines between different visits within control group.

Cytokines	Visit 1 N = 29	Visit 2 N = 29	Visit 3 N = 29
IL-1α	26.1 ±13.2	28.0 ±15.7	22.3 ±12.0
IL-1β	24.9 ±17.0	19.9 ±16.5	33.1 ±16.8
IL-2	11.3 ±6.3	8.6 ±3.9	10.9 ±5.9
IL-6	21.2 ±19.8	20.6 ±25.0	35.1 ±27.5*
IL-13	8.2 ±7.4	4.9 ±4.2	10.0 ±6.5
MCSF	21.2 ±8.9	25.8 ±9.2	27.9 ±7.5**
TNF-α	12.4 ±6.6	11.8 ±8.5	10.4 ±4.4
TNF-β	15.9 ±7.3	15.3 ±6.5	14.9 ±5.6
MCP-1	26.9 ±16.8	38.6 ±24.5***	46.6 ±22.4**
MIP-1α	14.4 ±5.9	14.9 ±4.4	15.3 ±5.6
Eotaxin-1	14.1 ±6.8	14.6 ±5.9	17.6 ±8.3

*P = 0.03 Visit 2 compared to Visit 3

**P<0.01 Visit 2 compared to Visit 3

***P = 0.04 Visit 1 compared to Visit 2

All comparisons are otherwise non-significant (P≥0.05)

IL, interleukin; MCSF, macrophage colony-stimulating factor; TNF, tumor necrosis factor; MCP, monocyte chemoattractant protein; MIP, macrophage inflammatory protein.

Data presented as pixel density percentage ± SD

## Discussion

There is an increased cytokine presence in vaginal washings in women at risk for preterm birth which changes following the use of 17-OHP. There is a paucity of human investigations surrounding 17-OHP use and the possible mechanisms of action in the reduction in preterm birth in at risk women. Our work focused on theimmune inflammatory changes in the vaginal environment in women utilizing 17-OHP. Results demonstrate an initial increased presence of interleukin-1 alpha, interleukin-1 beta, interleukin-2, and interleukin-13 in women with a prior preterm birth compared to controls. Interleukin-1 was the first pro-inflammatory cytokine to be associated with infection-mediated spontaneous preterm birth [12–16]. Imseis et al established that vaginal levels of interleukin-1were significantly elevated in laboring patients as compared with non-laboring patients [17]. Additionally, Romero et al identified that interleukin-1 alpha and interleukin-1 beta were significantly increased in patients with preterm premature rupture of membrane and preterm labor [18]. In the later third trimester, after 17-OHP use, interleukin-1 alpha, interleukin-1 beta, interleukin-2, and interleukin-13 presence matched the healthy controls. Similar to Monsanto et al, when comparing women with cervical insufficiency with control women, cytokines measured in the vaginal fluids were significantly higher in the patients before undergoing cerclage placement, and after the intervention, the cytokines levels of interleukin-1 beta, interleukin-6, interleukin-12, and MCP-1, and TNF-alpha became equivalent to that of control women at the time of admission [9]. Our findings confirm that one of the proposed mechanisms of 17-OHP reduction in preterm birth is through decreasing the cytokine inflammatory response generated by the fetal membranes and placenta [19].

In women at risk for preterm birth, the densities for eotaxin-1, MCP-1 and MIP-1 alpha cytokines were significantly suppressed when compared with healthy women. Eotaxin-1, which is also known as C-C motif chemokine-11, has been associated with recruitment of eosinophils by inducing their chemotaxis, and is involved in allergic responses [20]. Kraus et al describe a suppression of serum eotaxin-1 and MCP-1 throughout pregnancy compared to the postpartum period [21]. The clinical implication of progesterone suppression of eotaxin-1 and MCP-1 by the third trimester is unclear. MIP-1 alpha, a chemotactic cytokine, is produced by macrophages and crucial in the inflammatory response. Dudley et al described MIP-1 alpha produced by the decidual cells plays an important role in infection associated preterm labor [22]. Romero et al also found elevated MIP-1 alpha levels in amniotic fluid samples of women with infection associated preterm labor [23].

One limitation in our study was the small sample size for both arms of the investigation; however, given the significance of the condition, we believe that this cohort still provides valuable evidence. Our unique approach to investigating the immune response following progesterone use did not allow for a pre-investigation sample size calculation, which is similar to other comparable investigations [9]. Although differences in cytokine densities were identified in our investigation, potentially a larger number of women in each of the cohort groups may result in alternative findings. In our population, progesterone was effective in having women deliver in the later third trimester. The hypothetical levels of maternal vaginal cytokine densities of a very preterm birth or a failed progesterone therapy birth, may look completely different. Another limitation was the use of semi-quantitative pixel density analysis. Although this methodology has been utilized in many cytokine related investigations, an exact quantitative value may provide a better understanding of the human vaginal cytokine milieu [11]. In a living human reproductive system, there are innumerable cellular and soluble mediators which interact amongst each other and with surrounding cells. While this study has certain limitations, it nonetheless presents interesting and novel results in an area that has been substantially understudied.

The precise mechanism of preterm labor is unknown and it is likely multifactorial. The pathophysiology which allows progesterone to work is also poorly understood. Yet, the use of progesterone in the prevention of preterm birth has been effective in the subpopulation of women at risk for recurrence. Based on our work, progesterone likely prevents preterm birth through alterations in the maternal immune system. We demonstrated pro-inflammatory cytokines, interleukin-1 beta, interleukin-1 alpha, interleukin-2, and interleukin-13, densities were significantly elevated in women with a prior preterm birth compared with controls. This was followed by a downregulation of these cytokines following weekly administration of intramuscular progesterone. Additionally, other cytokines, eotaxin-1, MCP-1 and MIP-1 alpha, were also reduced following progesterone therapy which have been implicated in the preterm labor process. Further investigation into the immunological changes resulting from progesterone use in the prevention of preterm birth is warranted.

## Supporting information

S1 Table Cytokine comparisons between visits. The cytokine pixel density percentage and standard deviation (STD) comparing preterm birth group and controls. Control women pixel density percentages are compared between visit 1 & 2 and visit 1 & 3 (Control Visits). Preterm birth group women pixel density percentages are compared between visit 1 & 2 and visit 1 & 3 (Progesterone Visits). Red shading signifies P<0.05.
(XLSX)
